# Effects of P:Ni Ratio on Methanol Steam Reforming on Nickel Phosphide Catalysts

**DOI:** 10.3390/molecules28166079

**Published:** 2023-08-16

**Authors:** Abdulrahman Almithn

**Affiliations:** Department of Chemical Engineering, College of Engineering, King Faisal University, Al Ahsa 31982, Saudi Arabia; aalmithn@kfu.edu.sa

**Keywords:** nickel phosphides, density functional theory, methanol decomposition, steam reforming, dehydrogenation, fuel cells

## Abstract

This study investigates the influence of the phosphorus-to-nickel (P:Ni) ratio on methanol steam reforming (MSR) over nickel phosphide catalysts using density functional theory (DFT) calculations. The catalytic behavior of Ni(111) and Ni_12_P_5_(001) surfaces was explored and contrasted to our previous results from research on Ni_2_P(001). The DFT-predicted barriers reveal that Ni(111) predominantly favors the methanol decomposition route, where methanol is converted into carbon monoxide through a stepwise pathway involving CH_3_OH* → CH_3_O* → CH_2_O* → CHO* → CO*. On the other hand, Ni_12_P_5_ with a P:Ni atomic ratio of 0.42 (5:12) exhibits a substantial increase in selectivity towards methanol steam reforming (MSR) relative to methanol decomposition. In this pathway, formaldehyde is transformed into CO_2_ through a sequence of reactions involving CH_2_O*→ H_2_COOH* → HCOOH* → HCOO* → CO_2_. The introduction of phosphorus into the catalyst alters the surface morphology and electronic structure, favoring the MSR pathway. However, with a further increase in the P:Ni atomic ratio to 0.5 (1:2) on Ni_2_P catalysts, the selectivity towards MSR decreases, resulting in a more balanced competition between methanol decomposition and MSR. These results highlight the significance of tuning the P:Ni atomic ratio in designing efficient catalysts for the selective production of CO_2_ through the MSR route, offering valuable insights into optimizing nickel phosphide catalysts for desired chemical transformations.

## 1. Introduction

Proton-exchange membrane (PEM) fuel cells, water splitting, new-generation batteries, and CO_2_ conversion hold great promise as cutting-edge energy technologies for the future [1,2,3,4,5,6]. However, PEM fuel cells face challenges with hydrogen storage [7,8,9]. Methanol steam reforming (MSR) shows great potential as a viable solution for addressing this challenge given its ability to generate hydrogen from liquid methanol, which offers advantages in terms of storage and handling compared to hydrogen gas [10,11,12]. MSR is a relatively simple process that can be integrated with PEM fuel cells, making it an attractive option for on-board hydrogen production [13,14]. Methanol can be used to produce hydrogen through two main reactions: methanol decomposition and steam reforming. In methanol decomposition, methanol is broken down into hydrogen and carbon monoxide via:

CH_3_OH → 2H_2_ + CO (1)

To prevent CO poisoning in Pt-based fuel cells and further enhance hydrogen production, the water–gas shift (WGS) reaction is often used to further convert carbon monoxide to carbon dioxide via:

CO + H_2_O → H_2_ + CO_2_ (2)

Steam reforming provides a more efficient route to produce hydrogen directly from methanol:

CH_3_OH + H_2_O → 3H_2_ + CO_2_ (3)

thus eliminating CO production while producing a high yield of hydrogen [13,15,16,17,18,19,20,21,22].

Cu-based catalysts have been considered the traditional catalysts for MSR [9], but they face challenges related to rapid deactivation and inadequate thermal stability [22,23,24]. Recent studies have investigated other metal-based catalysts, such as Pd/ZnO and PdZn alloys [18,19], that have shown higher selectivity towards CO_2_ but lower catalytic activity [25,26,27,28]. The primary goal of catalyst performance enhancement for MSR is to increase the rate of hydrogen production and improve CO_2_ selectivity while using a thermally stable material. Transition metal phosphides (TMPs) have emerged recently as a highly versatile class of catalysts which can be used for a wide range of applications due to their unique selectivity and resistance to coke formation [29,30,31,32,33]. Among those TMPs, nickel phosphides (Ni*_x_*P*_y_*) have been previously examined for C–O bond cleavage in biomass-derived molecules [34,35]. These studies show that the addition of P atoms to Ni promotes cleaving the tertiary bond (^3^C–O) in 2-methyltetrahydrofuran relative to pure Ni in which cleaving the secondary bond (^2^C–O) is much more facile. Increasing the P:Ni atomic ratio (i.e., Ni → Ni_12_P_5_ → Ni_2_P) increases the selectivity towards ^3^C–O bond cleavage. The geometric or electronic effects caused by the incorporation of P atoms with Ni appear to play a role in this unique selectivity to activate hindered C–O bonds [36].

**Scheme 1:**
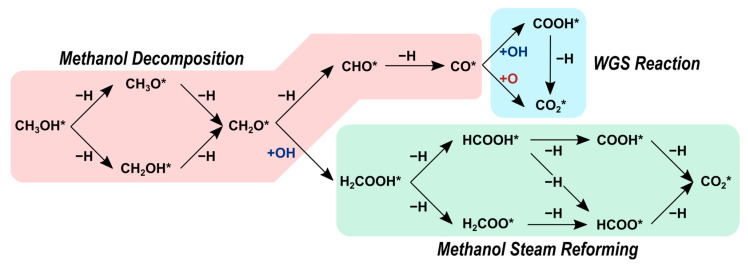
Reactions network to form CO and CO_2_ examined in our prior and current studies over Ni, Ni_12_P_5_, and Ni_2_P catalysts. Asterisk (*) denotes a surface site. Reprinted from reference [32].

In our previous study, we explored the reaction network shown in Scheme 1 over the Ni_2_P(001) surface using density functional theory (DFT) calculations [32]. Our DFT-derived activation barriers indicate that methanol decomposition on the Ni_2_P(001) surface occurs through CH_3_OH* → CH_3_O* → CH_2_O* → CHO* → CO*. In the MSR pathway, formaldehyde (CH_2_O*) reacts with a hydroxyl (OH*) originating from a water splitting reaction to yield H_2_COOH*, which ultimately produces CO_2_ via H_2_COOH*→ HCOOH* → HCOO* → CO_2_. The activation barriers for both routes are comparable (a difference of only 5 kJ mol^−1^) whereas, on transition metal catalysts, methanol decomposition is more likely to dominate. The role of phosphorus and the effects of the P:Ni atomic ratio on selectivity, however, remain unclear.

In this paper, we build upon our previous study by incorporating Ni and Ni_12_P_5_ catalysts to illustrate the notable impact of the P:Ni atomic ratio (*y*:*x* in Ni*_x_*P*_y_*) on the selectivity and reactivity of MSR. The results demonstrate that the choice of P:Ni atomic ratio plays a crucial role in shaping catalytic performance. The moderate P:Ni of 0.42 for Ni_12_P_5_ (5:12) enhances the selectivity of MSR relative to methanol decomposition. The insights gained from these findings can guide the design of efficient catalysts tailored for use in MSR applications.

## 2. Results and Discussion

### 2.1. Optimized Adsorbates and Their Binding Energies

In this section, we study the geometries and energies of all the intermediates that form during methanol decomposition and MSR. We aim to identify the most stable configuration by exploring various adsorption modes on examined surfaces, as depicted in Figures S1 and S2 (Supplementary Materials). The binding energies presented in Table 1 were calculated using Equation (4).

**Figure 1:**
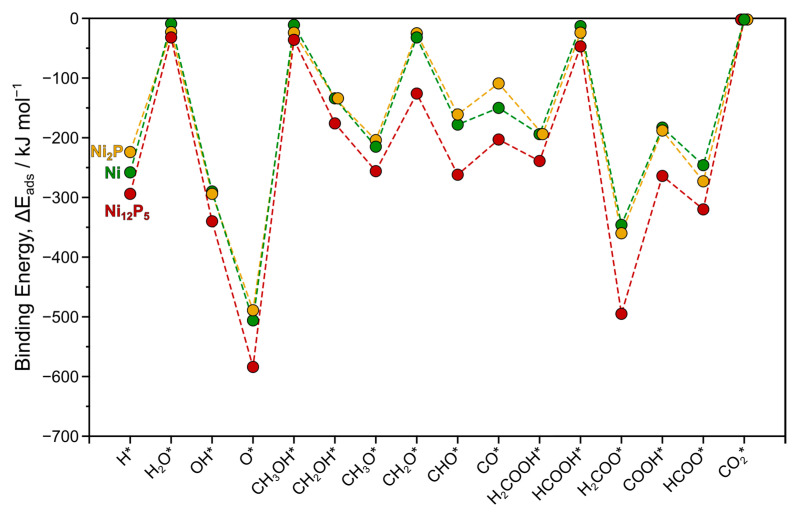
Trends in binding energies Δ*E_ads_* (kJ mol^−1^) for all surface intermediates examined in this study over Ni (green) and Ni_12_P_5_ (red) surfaces compared to Ni_2_P (yellow) examined in our previous study [32]. Dashed lines are provided as visual guides. Asterisk (*) denotes a surface site.

Methanol (CH_3_OH) binds weakly to the atop site (M_1_) of the Ni(111) surface through its oxygen atom (Figure S1e), with a binding energy of −11 kJ mol^−1^ (Table 1). This observation aligns with those found in previous theoretical and experimental studies, which also demonstrate the weak adsorption of methanol onto Ni and other transition metals [37,38,39,40]. Similarly, other physisorbed species such as water and formic acid also exhibit low binding energy to the atop site (−9 and −13 kJ mol^−1^, respectively). The interaction between these three intermediates and the surface metal atom likely involves the donation of the oxygen atom’s lone pair of electrons, contributing to their adsorption. Formaldehyde (CH_2_O; Figure S1h), on the other hand, displays a slightly stronger binding energy (−32 kJ mol^−1^) and preferentially binds to the 3-fold site (M_3_) in a top-bridge configuration. While it can also bind to the bridging site (M_2_) in a di-σ mode, this configuration is less favored by 18 kJ mol^−1^. The remaining unsaturated intermediates demonstrate stronger binding energies ranging from −134 kJ mol^−1^ for CH_2_OH* to −506 kJ mol^−1^ for O* (Table 1). The majority of these intermediates (H*, OH*, O*, CH_3_O*, CHO*, CO*, H_2_COOH*, and H_2_COO*) prefer to adsorb on the 3-fold site (M_3_) except CH_2_OH*, COOH*, and HCOO* which favorably bind to the bridging site (M_2_). The trends in binding energies observed here on Ni(111) closely resemble those seen in our previous study on the Ni_2_P(001) surface (Figure 1).

Compared to the Ni(111) and Ni_2_P(001) surfaces, the Ni_12_P_5_(001) surface exhibits an overall increase in binding strength for all surface intermediates (Figure 1). While the differences in binding energies among different species remain generally similar, the average binding energy on Ni_12_P_5_(001) is notably higher, with an average binding energy of approximately −230 kJ mol^−1^ on Ni_12_P_5_, compared to −170 kJ mol^−1^ on both Ni(111) and Ni_2_P(001). For example, methanol, water, and formic acid also bind to the metal atop site (M_1_), but with stronger affinities relative to Ni and Ni_2_P surfaces. Formaldehyde, a crucial intermediate in the MSR pathway, exhibits a binding energy of −126 kJ mol^−1^ when interacting with the metal 4-fold site on Ni_12_P_5_(001), which is over 100 kJ mol^−1^ stronger than its binding energy on Ni_2_P(001). This is consistent with the differences in calculated binding energies on Ni, Ni_12_P_5_, and Ni_2_P reported in previous studies for the intermediates involved in the hydrodeoxygenation reaction of oxygenated compounds [34]. Ni_12_P_5_(001) has two unique 4-fold sites denoted by M_4a_ and M_4b_ (discussed in Section 3). Notably, the M_4b_ site displays higher reactivity compared to M_4a_, as evidenced by the preference of most intermediates examined here, with the exception of H_2_COOH*. For instance, formaldehyde (CH_2_O) binds more strongly to the M_4b_ site by around 75 kJ mol^−1^ compared to the M_4a_ site. These findings collectively suggest that Ni_12_P_5_ exhibits higher reactivity compared to Ni and Ni_2_P surfaces.

### 2.2. Reaction Pathways

Next, we present a thorough analysis of the reaction pathways shown in Scheme 1 on Ni and Ni_12_P_5_. We compare these results with our prior work on Ni_2_P to elucidate the effects of the P:Ni atomic ratio on selectivity and reactivity [32]. The reaction network, as illustrated in Scheme 1, serves as the basis for our investigation. To ensure that the most stable transition states are identified, we extensively explored various configurations on distinct surface sites for each elementary step. Table 2 displays the respective forward activation barriers and reaction energies. These values do not encompass the diffusion steps, as these steps are typically assumed to be relatively fast and have negligible influence on the overall reaction pathways.

#### 2.2.1. H_2_O Dissociation

On the Ni(111) surface, the direct water dissociation (reaction 17; Figure S3) has an enthalpic activation barrier (Δ*H*_act_) of 74 kJ mol^−1^ and is an exothermic reaction with a reaction energy (Δ*H*_rxn_) of −25 kJ mol^−1^ (reaction 17; Table 2). Further dehydrogenation of hydroxyl (OH*) adsorbed onto the M_3_ site takes place with a higher activation barrier of 97 kJ mol^−1^ and a reaction energy of −18 kJ mol^−1^. These values agree with previously reported data on the Ni(111) surface [41], and they are consistent with our earlier findings on the Ni_2_P(001) surface, where OH* activation demonstrated a larger barrier. The disproportion of two adjacent OH* species can produce O* and H_2_O* with an activation barrier of only 28 kJ mol^−1^. Our previous study revealed that H_2_O* dissociation can be promoted by hydrogen bonding with another vicinal H_2_O* molecule. Here, on Ni(111), we observe a slight decrease in the activation barrier of H_2_O-assisted water dissociation (reaction 20; Table 2) by 6 kJ mol^−1^. The structures of the reactants, transition states, and products for all reactions examined in this study are shown in Figures S3 and S4 (Supplementary Materials).

On Ni_12_P_5_(001), we observe a similar trend, albeit with lower activation barriers compared to both Ni(111) and Ni_2_P(001) surfaces. For example, H_2_O* dissociates with an activation barrier of 49 kJ mol^−1^ (reaction 17; Table 2), which is 25 and 42 kJ mol^−1^ lower than the calculated barriers on Ni and Ni_2_P, respectively. Concerted water dissociation (reaction 20) is also more facile, with an activation barrier of only 35 kJ mol^−1^. Thus, we believe that hydrogen bonding plays an important role in H_2_O dissociation over nickel phosphide catalysts.

#### 2.2.2. Methanol Decomposition

**Figure 2:**
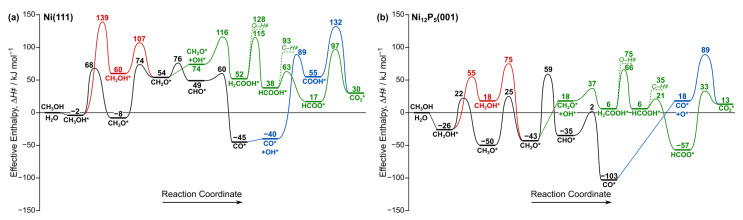
Effective enthalpy (Equation (5); 573 K) diagram for the reactions network shown in Scheme 1 on (**a**) Ni(111) and (**b**) Ni_12_P_5_(001). Abstracted H atoms are desorbed as H_2_(g). Unfavorable routes are displayed using dashed lines. A similar diagram for Ni_2_P(001) is shown in Figure S5 (Supplementary Materials). Asterisk (*) denotes a surface site. Black and red represent methane decomposition pathway, green represents MSR pathway, and blue represents WGS reaction pathway.

Methanol (CH_3_OH) can either undergo C–H or O–H bond activation (reactions 1 and 2; Table 2) on the Ni(111) surface. However, the activation barrier of the C–H bond to form CH_2_OH* (141 kJ mol^−1^) is twice that of O–H bond activation required to form CH_3_O* (70 kJ mol^−1^), suggesting that the methoxy formation route is more favorable. Similarly, on the Ni_12_P_5_(001) surface, O–H activation in methanol is more favorable by 33 kJ mol^−1^. In order to assess the relative preference of the various reaction routes shown in Scheme 1, we next analyze the effective enthalpy barriers (Δ*H*҂; Equation (5)) as shown in Figure 2. These Δ*H*҂ values are referenced to the energies of gas-phase methanol and water, which is particularly relevant when the reaction involves water.

On the Ni(111) surface, the O–H bond of physisorbed CH_3_OH* is initially cleaved to from CH_3_O* with an effective barrier (Δ*H*҂) of 68 kJ mol^−1^ (Figure 2a). A reaction cleaving the C–H bond in CH_3_O* then requires a barrier of 74 kJ mol^−1^ to form formaldehyde (CH_2_O*), and the reverse path to form CH_3_O* requires only 20 kJ mol^−1^. Methoxy is a stable intermediate relative to other intermediates in the methanol decomposition pathway, as shown in Figure 2a. This suggests that CH_3_O* is more likely the dominant species on Ni(111) as reported in previous experimental studies [42,43]. Subsequent dehydrogenation of CH_2_O* leads to the formation of CHO* and finally CO* with activation barriers of 76 and 60 kJ mol^−1^, respectively. For Ni_12_P_5_(001), the methanol decomposition steps followed to form CH_3_O* and then CH_2_O* are both facile, with relatively similar activation barriers (22–25 kJ mol^−1^; Figure 2b). Once CH_2_O* is formed, it can undergo C–H bond activation to form CHO* with a barrier of 59 kJ mol^−1^, which eventually forms CO* with a barrier of only 2 kJ mol^−1^. Both CH_3_O* and CH_2_O* are stable intermediates on Ni_12_P_5_, in contrast to Ni and Ni_2_P, where CH_3_O* is more dominant.

#### 2.2.3. Methanol Steam Reforming

**Figure 3:**
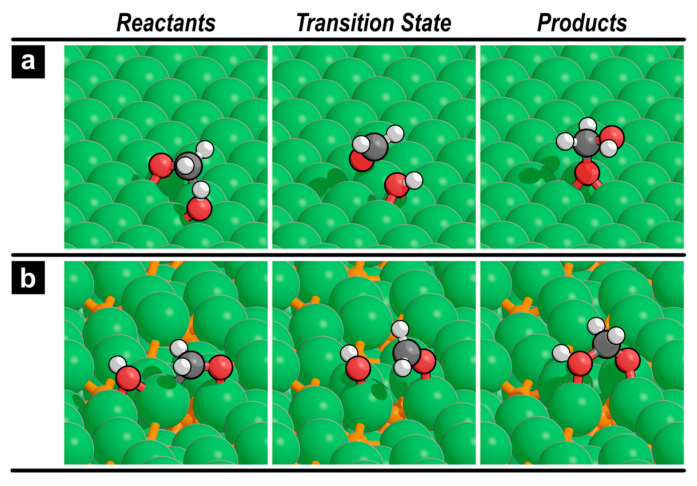
Reactants, transition state, and products for CH_2_O* reaction with OH* (reaction 7) on (**a**) Ni(111) and (**b**) Ni_12_P_5_(001) surfaces.

Here, we examine the possibility of reacting formaldehyde (CH_2_O*) with co-adsorbed OH* to form H_2_COOH*. On the Ni(111) surface, this reaction requires an effective activation barrier of 116 kJ mol^−1^ (Figure 2a), which is much larger than the activation barrier of further decomposition to CHO* (76 kJ mol^−1^). This is consistent with the results of the previous studies that demonstrated the preference of transition metal catalysts to decompose methanol into CO, except for Cu, which shows a distinct selectivity towards H_2_COOH* formation via MSR. In contrast, Ni_12_P_5_(001) can form H_2_COOH* from CH_2_O* and OH* with a barrier of 37 kJ mol^−1^ (Figure 2b), which is 22 kJ mol^−1^ lower than that of CH_2_O* decomposition. In our previous study, we found that the barriers of CH_2_O* decomposition to CHO* and CH_2_O* reaction with OH* are within ~5 kJ mol^−1^ on the Ni_2_P(001) surface (Figure S5), indicating that both pathways are kinetically relevant. Figure 3 shows the reactant, transition state, and product structures for reaction 7 on Ni(111) and Ni_12_P_5_(001) surfaces. On both surfaces, the carbon atom in CH_2_O* needs to undergo partial desorption from the surface before reacting with the adjacent OH* to form H_2_COOH*.

Once H_2_COOH* is formed on the Ni_12_P_5_(001) surface, it can either cleave its C–H bond or its O–H bond, but C–H bond cleavage with an activation barrier of 66 kJ mol^−1^ is slightly more favorable (Figure 2b). Formic acid (HCOOH*) then forms HCOO*, which eventually forms CO_2_*, the product of the MSR reaction. Taken together, these findings indicate that the P:Ni atomic ratio can dictate the selectivity towards CO_2_ formation via MSR relative to methanol decomposition into CO. The moderate P:Ni atomic ratio in Ni_12_P_5_ (P:Ni = 0.42) favors the MSR pathway compared to Ni_2_P (P:Ni = 0.5), in which both methanol decomposition and MSR are competitive. Ni (P:Ni = 0), on the other hand, predominantly decomposes methanol to carbon monoxide.

The impact of the P:Ni atomic ratio on the differences in selectivity stems from a complex interplay between geometric and electronic factors, each contributing to the observed effects. For example, the Ni atoms exposed on the Ni_2_P(001) surface form 3-fold sites similar to those found in Ni(111), only with larger Ni–Ni bond distances (3.16 Å versus 2.49 Å). This may explain the similarities in the reaction coordinate diagram between Ni (Figure 2a) and Ni_2_P (Figure S5), except that both MSR and methanol decomposition pathways start to compete on Ni_2_P, whereas methanol decomposition is more dominant on Ni. The Ni_12_P_5_(001) surface, however, exposes unique metal 4-fold sites (M_4_) that predominantly favor MSR. Another factor to consider here is the electronic effects. We have previously performed charge analysis for these three surfaces and we have shown that the Ni atoms become more positively charged with an increasing P:Ni atomic ratio [35]. This may indicate that the moderate Lewis acidity on Ni_12_P_5_ enhances the selectivity of MSR relative to methanol decomposition.

#### 2.2.4. Water–Gas Shift Reaction

CO* generated through the methanol decomposition route can be transformed into CO_2_ via the water–gas shift reaction. It can either react with a co-adsorbed OH* to form COOH* in the carboxyl mechanism, which subsequently dehydrogenates to produce CO_2_ (CO* + OH* → COOH* → CO_2_* + H*), or it can react with O* to directly produce CO_2_ via the redox mechanism (CO* + O* → CO_2_*), as shown in Scheme 1. The carboxyl mechanism was found to be more favorable on Ni_2_P than the redox mechanism, which is similar to what we found here on the Ni(111) surface (Table 2). However, for Ni_12_P_5_(001), we could not find a stable transition state for the carboxyl mechanism. Instead, Ni_12_P_5_ exclusively forms CO_2_ via the redox mechanism with a large effective barrier of 89 kJ mol^−1^ (Figure 2b). WGR reaction pathways involve substantially higher barriers than those observed for the MSR pathway across all catalysts studied here. Thus, CO_2_ formation via the WGS reaction can be ruled out.

## 3. Computational Methods

All periodic DFT calculations were carried out using the Vienna ab initio simulation package (VASP) [44,45,46,47] and employing the computational catalysis interface (CCI) [48]. Projector augmented-wave (PAW) potentials with an energy cutoff of 396 eV were used to construct the plane waves [49,50]. The exchange and correlation energies were described using the revised Perdew–Burke–Ernzerhof (RPBE) form of the generalized gradient approximation [51,52,53]. For modeling gaseous species, 15 × 15 × 15 Å unit cells were used. Available crystallographic data were used to obtain the bulk unit cells of Ni and Ni_12_P_5_, which were then optimized using DFT to determine their lattice parameters, as reported in more detail in our previous work [34]. Spin polarization was used for all Ni calculations, whereas Ni_12_P_5_ does not exhibit ferromagnetic properties.

**Figure 4:**
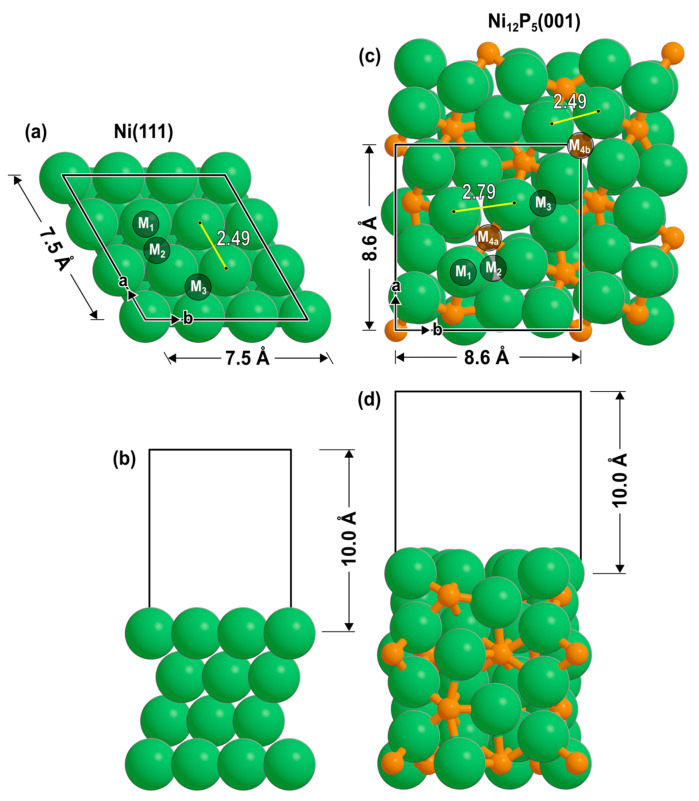
(**a**,**b**) Ni(111) and (**c**,**d**) Ni_12_P_5_(001) slabs as seen from top and side views. Labels denote the binding sites (M_1_: atop; M_2_: bridge; M_3_: 3-fold hollow; M_4_: 4-fold hollow).

A 3 × 3 periodic lattice was used to model the Ni(111) surface (Figure 4a), with four atomic layers and 10 Å of vacuum orthogonal to the surface (Figure 4b). For geometric convergence, a *k*-point mesh of 3 × 3 × 1 was employed [54,55]. This was followed by a single-point calculation with a *k*-point mesh of 6 × 6 × 1 to compute the electronic energy. For the Ni_12_P_5_ catalyst, the Ni-terminated (001) surface was used (Figure 4c) due to its favorable surface formation energy, as shown previously [34], with two repeating units (8 atomic layers) and 10 Å of vacuum orthogonal to the surface (Figure 4d). This termination does not expose any phosphorus atoms on the surface. It has atop sites (M_1_), bridging sites (M_2_), 3-fold sites (M_3_), and two unique 4-fold sites (M_4a_ and M_4b_). The M_4a_ site has a P atom directly beneath it (in the second layer), whereas the P atom beneath M_4b_ is in the fourth layer. A *k*-point mesh of 3 × 3 × 1 was used for Ni_12_P_5_ calculations. The bottom half of each catalyst model was constrained during optimizations.

Transition state structures were identified using the combination of the nudged elastic band (NEB) method and the dimer method [56,57,58]. Electronic energies converged to within 10^−6^ eV, and forces converged to less than 0.05 eVÅ^−1^. Frequency calculations were performed, with all catalysts’ atoms constrained, to estimate enthalpies at 573 K. The binding energy (Δ*E_ads_*) is defined as:

(4) $$∆E_{ads}=E_{species/surf\;}-E_{species(g)}-E_{surf}$$

and effective enthalpy barriers (Δ*H*҂) are calculated using:

(5) $$∆H҂=H҂+\lambda H_{H_{2}(g)}-H_{CH_{3}OH(g)}-H_{H_{2}O(g)}-H_{surf}$$

where *λ* is the number of H_2_ molecules desorbed from the surface as a result of the hydrogen removal steps. Section S1 in the Supplementary Materials provides more details regarding the computational methods.

## 4. Conclusions

The effects of the P:Ni ratio on selectivity towards the methanol steam reforming reaction were investigated using periodic density functional theory calculations. These calculations showed that Ni(111) surface (P:Ni = 0) predominantly decomposes methanol into carbon monoxide through: CH_3_OH* → CH_3_O* → CH_2_O* → CHO* → CO*. These results are in agreement with those of prior studies on various transition metals. On the other hand, Ni_12_P_5_(001) with a P:Ni atomic ratio of 0.42 favors the methanol steam reforming pathway of CO_2_ production from formaldehyde via CH_2_O* + OH* → H_2_COOH*→ HCOOH* → HCOO* → CO_2_. Increasing the P:Ni atomic ratio to 0.5 in Ni_2_P(001) decreases the selectivity of MSR relative to methanol decomposition, but both pathways remain competitive. The catalytic surfaces analyzed in this study displayed negligible activity towards WGS reaction pathways, as indicated by the substantial activation barriers observed. These findings shed light on the catalytic behavior of nickel phosphide catalysts and provide valuable insights into the distinctive pathways of CO_2_ generation, guiding the development of efficient and selective catalytic materials for MSR.

## Figures and Tables

**Table 1:** Binding energies Δ*E_ads_* (Equation (4); kJ mol^−1^) of examined intermediates and their preferred adsorption configuration on Ni(111), Ni_12_P_5_(001), and Ni_2_P(001).

	Ni(111)		Ni_12_P_5_(001)		Ni_2_P(001) [32]	
Species	Adsorption Mode	Δ*E_ads_*	Adsorption Mode	Δ*E_ads_*	Adsorption Mode	Δ*E_ads_*
		kJ mol^−1^		kJ mol^−1^		kJ mol^−1^
H*	M_3_	−258	M_4b_	−294	M_3_	−224
H_2_O*	M_1_	−9	M_1_	−32	M_1_	−23
OH*	M_3_	−290	M_4b_	−340	M_3_	−294
O*	M_3_	−506	M_4b_	−584	MP	−489
CH_3_OH*	M_1_	−11	M_1_	−36	M_1_	−24
CH_2_OH*	M_2_	−134	M_2_	−176	M_2_	−133
CH_3_O*	M_3_	−215	M_4b_	−256	M_3_	−204
CH_2_O*	M_3_	−32	M_4b_	−126	M_3_	−25
CHO*	M_3_	−178	M_4b_	−262	M_2_	−161
CO*	M_3_	−150	M_4b_	−203	M_1_	−109
H_2_COOH*	M_3_	−194	M_4a_	−239	M_3_	−194
HCOOH*	M_1_	−13	M_1_	−47	M_1_	−24
H_2_COO*	M_3_	−346	M_4b_	−495	M_3_	−360
COOH*	M_2_	−183	M_4b_	−264	M_2_	−188
HCOO*	M_2_	−246	M_2_	−320	M_2_	−273
CO_2_*	Parallel	−2	Vertical	−2	Parallel	−1

Asterisk (*) denotes a surface site.

**Table 2:** Forward activation enthalpy (Δ*H*_act_ = *H*_TS_ − *H*_reactants_) and the reaction energy (Δ*H*_rxn_ = *H*_products_ − *H*_reactants_) in kJ mol^−1^ for the elementary reactions analyzed in this study at 573 K.

No.	Reaction	Ni(111)		Ni_12_P_5_(001)		Ni_2_P(001) [32]	
		Δ*H*_act_	Δ*H*_rxn_	Δ*H*_act_	Δ*H*_rxn_	Δ*H*_act_	Δ*H*_rxn_
1	CH_3_OH → CH_2_OH + H	141	25	81	−4	114	63
2	CH_3_OH → CH_3_O + H	70	−36	48	7	98	9
3	CH_2_OH → CH_2_O + H	47	−30	57	−98	82	−8
4	CH_3_O → CH_2_O + H	82	42	75	−20	73	41
5	CH_2_O → CHO + H	22	−43	39	−52	17	−3
6	CHO → CO + H	11	−128	37	−105	19	−60
7	CH_2_O + OH → H_2_COOH	42	−18	18	−4	5	−40
8	H_2_COOH → HCOOH + H	63	−50	54	−56	33	−38
9	H_2_COOH → H_2_COO + H	72	0	69	−29	87	43
10	HCOOH → COOH + H	55	−20	27	−59	82	11
11	HCOOH → HCOO + H	25	−58	15	−108	61	20
12	H_2_COO → HCOO + H	23	−124	64	−61	13	−64
13	HCOO → CO_2_ + H	80	−24	89	43	39	−33
14	COOH → CO_2_ + H	81	−59	71	−10	115	−25
15	CO + OH → COOH	129	95	-	-	69	53
16	CO + O → CO_2_	144	56	71	13	128	3
17	H_2_O → OH + H	74	−25	49	−45	91	5
18	OH → O + H	97	−18	98	−57	161	81
19	OH + OH → H_2_O + O	28	−20	55	4	63	55
20	2H_2_O → H_2_O + OH + H	68	−4	35	−64	68	23

## Data Availability

Not applicable.

## Supplementary Materials

The following supporting information can be downloaded at: https://www.mdpi.com/article/10.3390/molecules28166079/s1, Section S1: Computation Methods Details; Figure S1: Images for adsorbed intermediates on the Ni(111) surface; Figure S2: Images for adsorbed intermediates on the Ni_12_P_5_(001) surface; Figure S3: Reaction images on the Ni(111) surface; Figure S4: Reaction images on the Ni_12_P_5_(001) surface; Figure S5: Effective enthalpy diagram for Ni_2_P(001).
