# Supplementary material for: Effects of P:Ni Ratio on Methanol Steam Reforming on Nickel Phosphide Catalysts
Source: Molecules. 2023 Aug 16;28(16):6079. doi: 10.3390/molecules28166079 (PMC10459788; doi:10.3390/molecules28166079)
Supplement: Supplementary file 1 [file molecules-28-06079-s001.zip › molecules-2559336-supplementary.pdf]

molecules

Supplementary Materials

Effects of P:Ni Ratio on Methanol Steam Reforming on Nickel Phosphide Catalysts

Abdulrahman Almithn

Department of Chemical Engineering, College of Engineering, King Faisal University, Al Ahsa 31982, Saudi Arabia; aalmithn@kfu.edu.sa

S1. Computation Methods Details

The unit cells of bulk nickel (with a space group of $Fm\bar{3}m$), Ni_2P (with a space group of $P\bar{6}2m$), and $Ni_{12}P_5$ (with a space group of $I4/m$) were constructed based on crystallographic data. Subsequently, the lattice parameters were optimized using DFT. Calculations for bulk nickel, $Ni_{12}P_5$, and Ni_2P involved the use of a Γ-centered 7 × 7 × 7 sampling of the Brillouin zone for Ni and an 8 × 8 × 14 sampling for $Ni_{12}P_5$ and Ni_2P. The optimized bulk lattice parameters were found to be in agreement with previous DFT studies and closely resembled the measured values. Specifically, the optimized lattice parameters for Ni were a = b = c = 3.52 Å, for Ni_2P they were a = b = 5.87 Å, c = 3.37 Å, and for $Ni_{12}P_5$ they were a = b = 8.63 Å, c = 5.07 Å.

Frequency calculations were performed on gas phase molecules and all optimized adsorbed species to determine zero-point vibrational energies (ZPVE), and vibrational, translational and rotational enthalpy and free energy. These terms were then used, together with electronic energies (E_0, provided by VASP), to estimate enthalpies (H)

$$H = E_0 + \mathrm{ZPVE} + H_{\mathrm{vib}} + H_{\mathrm{trans}} + H_{\mathrm{rot}} \quad \text{(S1)}$$

and free energies (G)

$$G = E_0 + \mathrm{ZPVE} + G_{\mathrm{vib}} + G_{\mathrm{trans}} + G_{\mathrm{rot}} \quad \text{(S2)}$$

for reactants, products, and transition states at 573 K (the typical temperature for MSR). For calculations which include a periodic metal surface, there are no translational or rotational degrees of freedom and DFT-derived vibrational frequencies can be used to determine the ZPVE, H_{vib}, and G_{vib}

$$ZPVE = \textstyle\sum_i (½\nu_i h) \quad \text{(S3)}$$

$$H_{vib} = \sum_i \left(\frac{\nu_i h e^{\frac{-\nu_i h}{kT}}}{1-e^{\frac{-\nu_i h}{kT}}} \right) \quad \text{(S4)}$$

$$G_{vib} = \sum_i \left(-kT \ln \frac{1}{1-e^{\frac{-\nu_i h}{kT}}} \right) \quad \text{(S5)}$$

For gaseous molecules, translational and rotational enthalpies and free energies were also computed from statistical mechanics:

$$H_{trans} = \frac{5}{2}kT \quad \text{(S6)}$$

$$H_{rot,linear} = kT \quad \text{(S7)}$$

$$H_{rot,nonlinear} = \frac{3}{2}kT \quad \text{(S8)}$$

$$G_{trans} = -kT \ln[\left(\frac{2\pi MkT}{h^2}\right)^{3/2} V] \quad \text{(S9)}$$

$$G_{rot} = -kT \ln[\frac{\pi^{1/2}}{\sigma}\left(\frac{T^3}{\theta_x\theta_y\theta_z}\right)^{1/2}] \quad \text{(S10)}$$

$$\theta_i = \frac{h^2}{8\pi^2 I_i k} \quad \text{(S11)}$$

where I_i is the moment of inertia about axes x, y or z and σ is the symmetry number of the molecule (2 for H_2 and 6 for C_2H_6). Equations S10–S12 obtained from: McQuarrie, D. A.; Statistical Mechanics; Sausolito, CA.

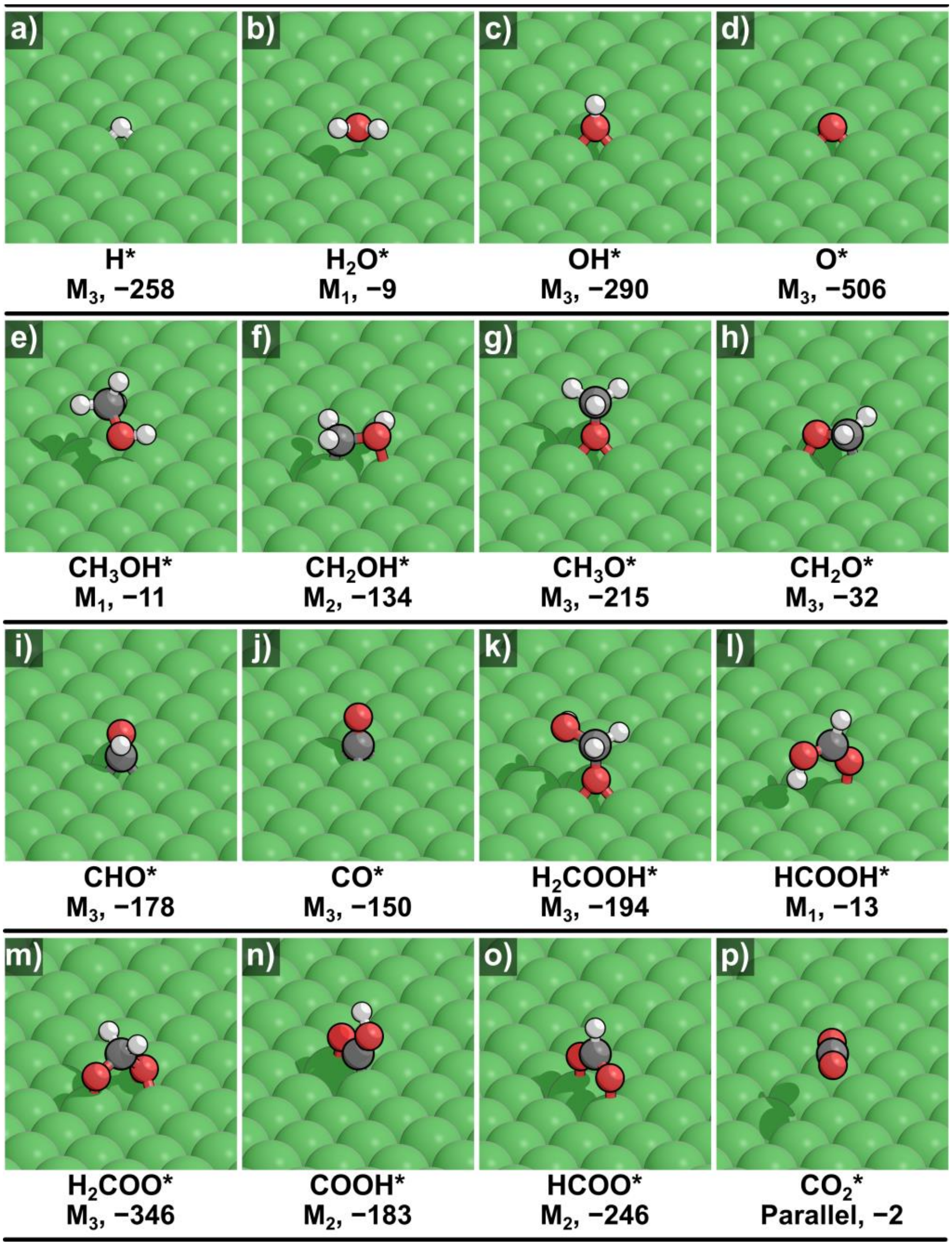

Figure S1. (a–p) The most stable adsorption geometries for all MSR intermediates on Ni(111) surface. Shown beneath each image are the adsorption mode and the binding energy in kJ mol^{-1}.

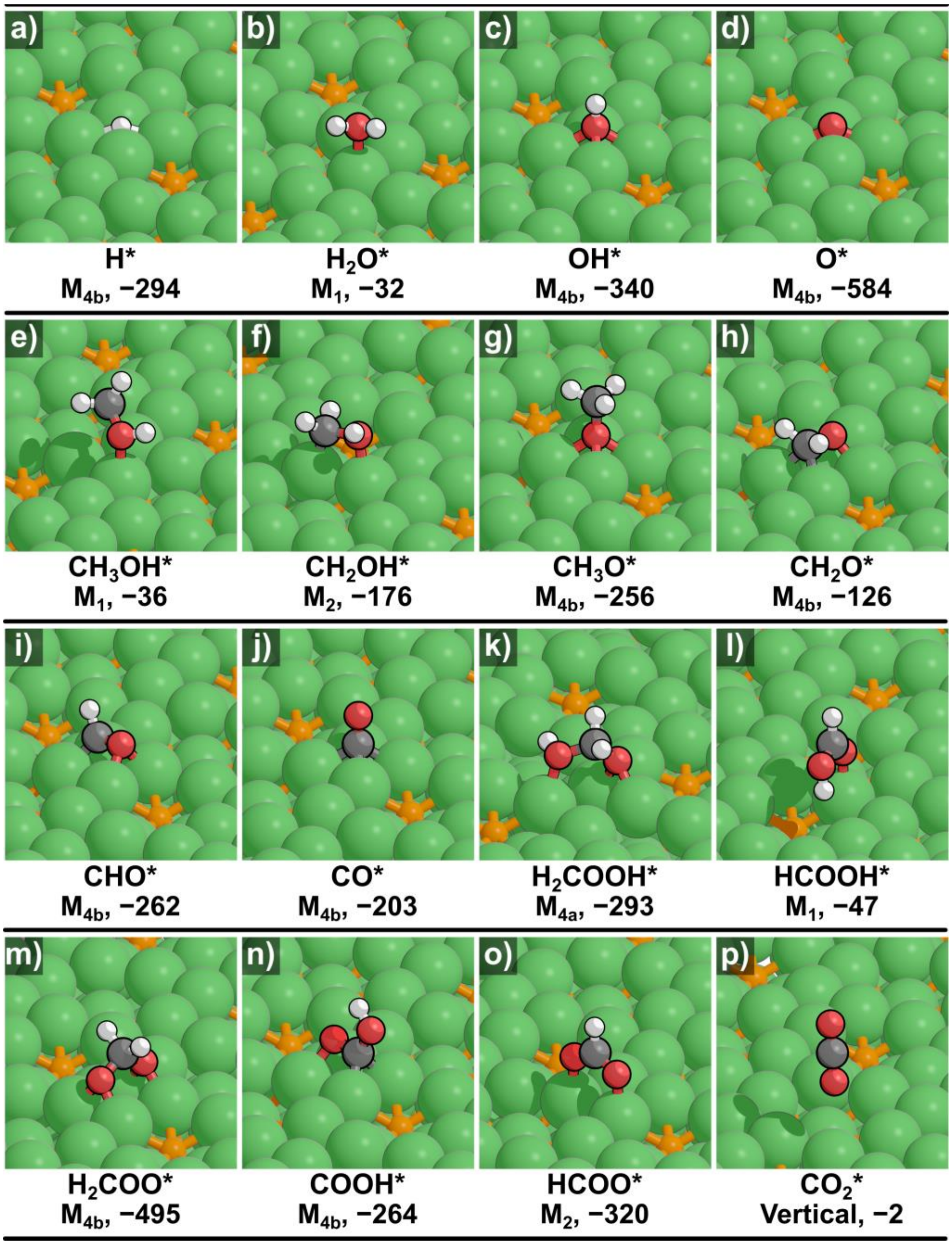

Figure S2. (a–p) The most stable adsorption geometries for all MSR intermediates on $Ni_{12}P_5$(001) surface. Shown beneath each image are the adsorption mode and the binding energy in kJ mol^{-1}.

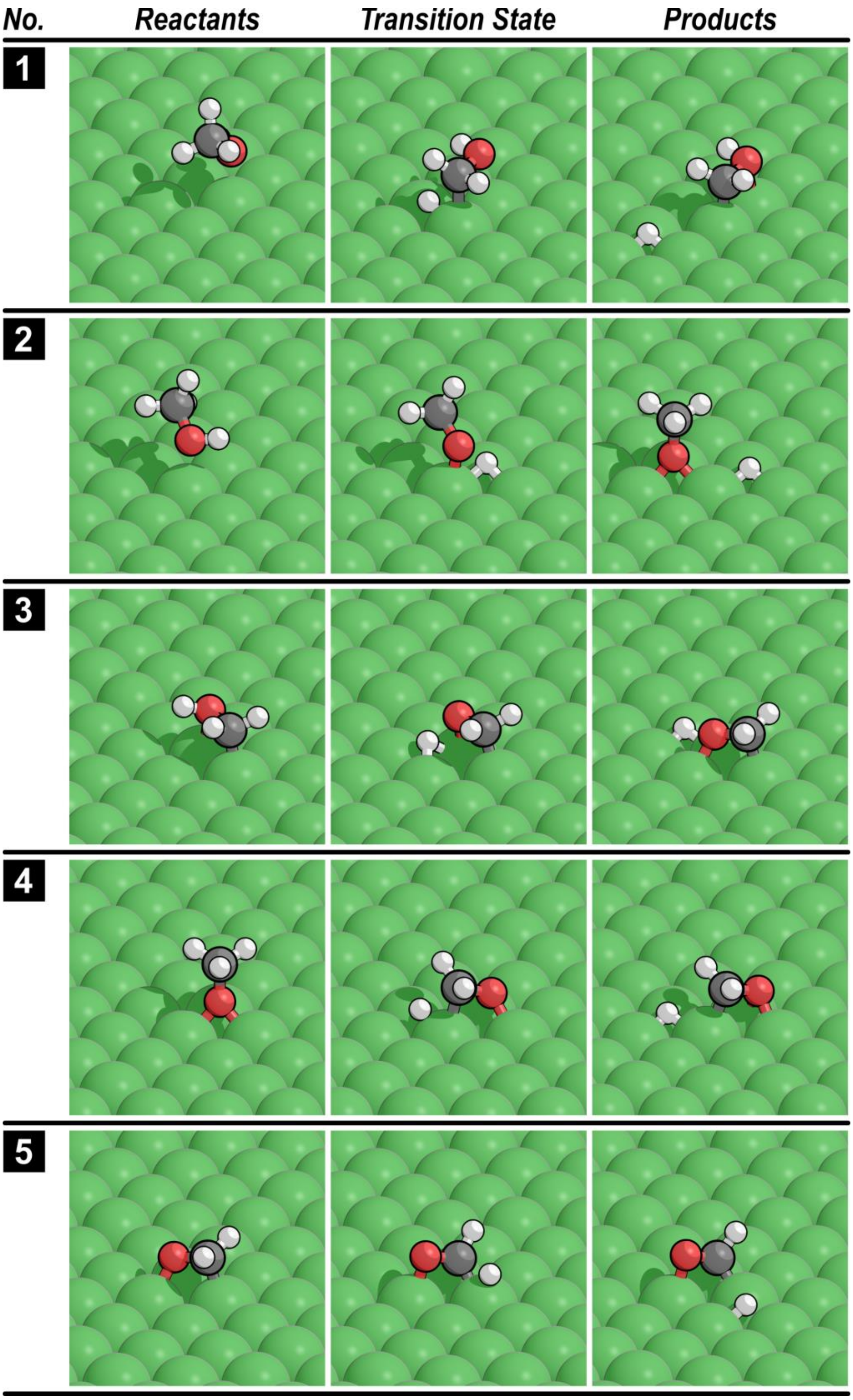

Figure S3. Reactants, transition state, and products structures for all reactions listed in Table 2 over Ni(111) surface.

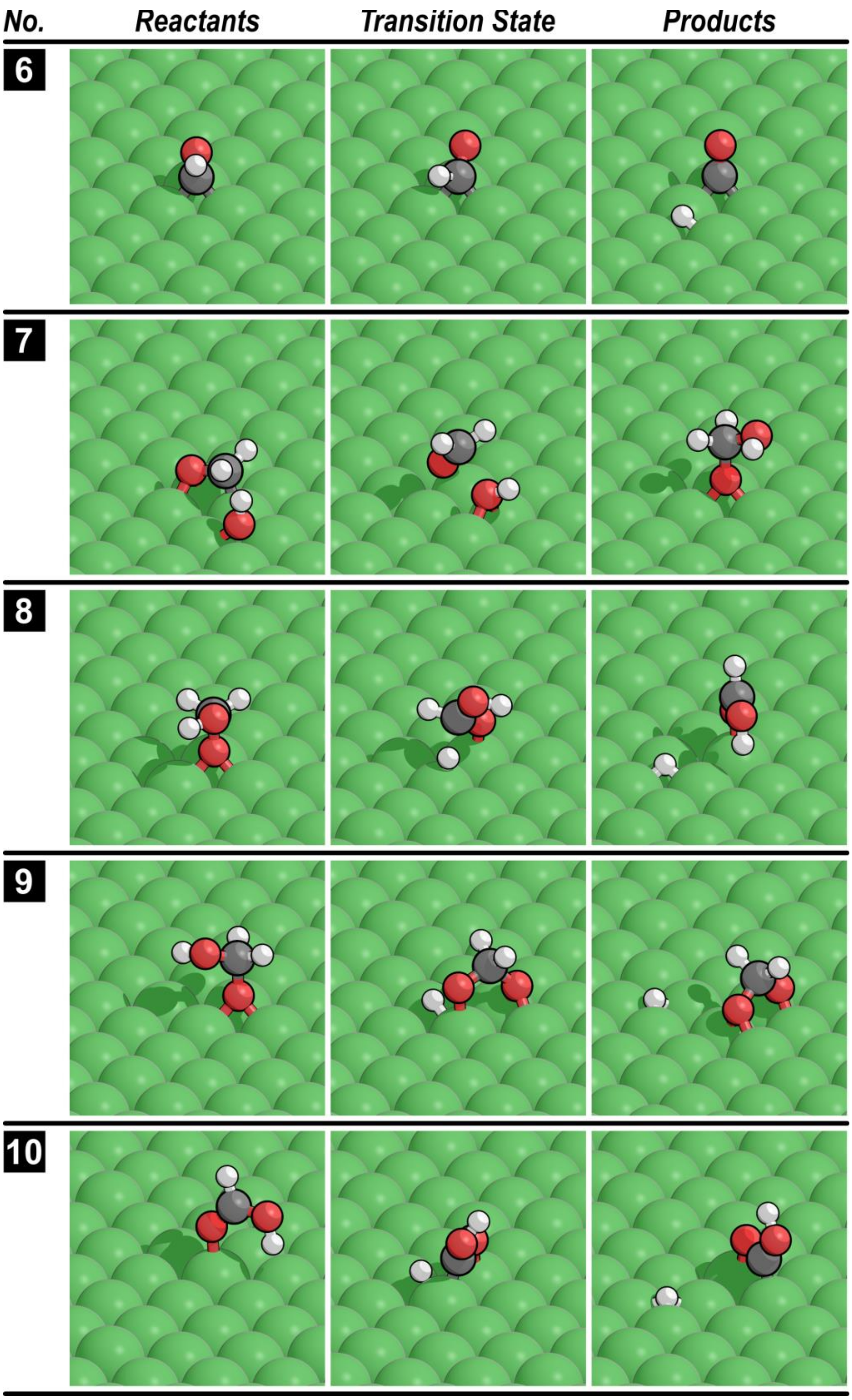

Figure S3 Cont. Reactants, transition state, and products structures for all reactions listed in Table 2 over Ni(111) surface.

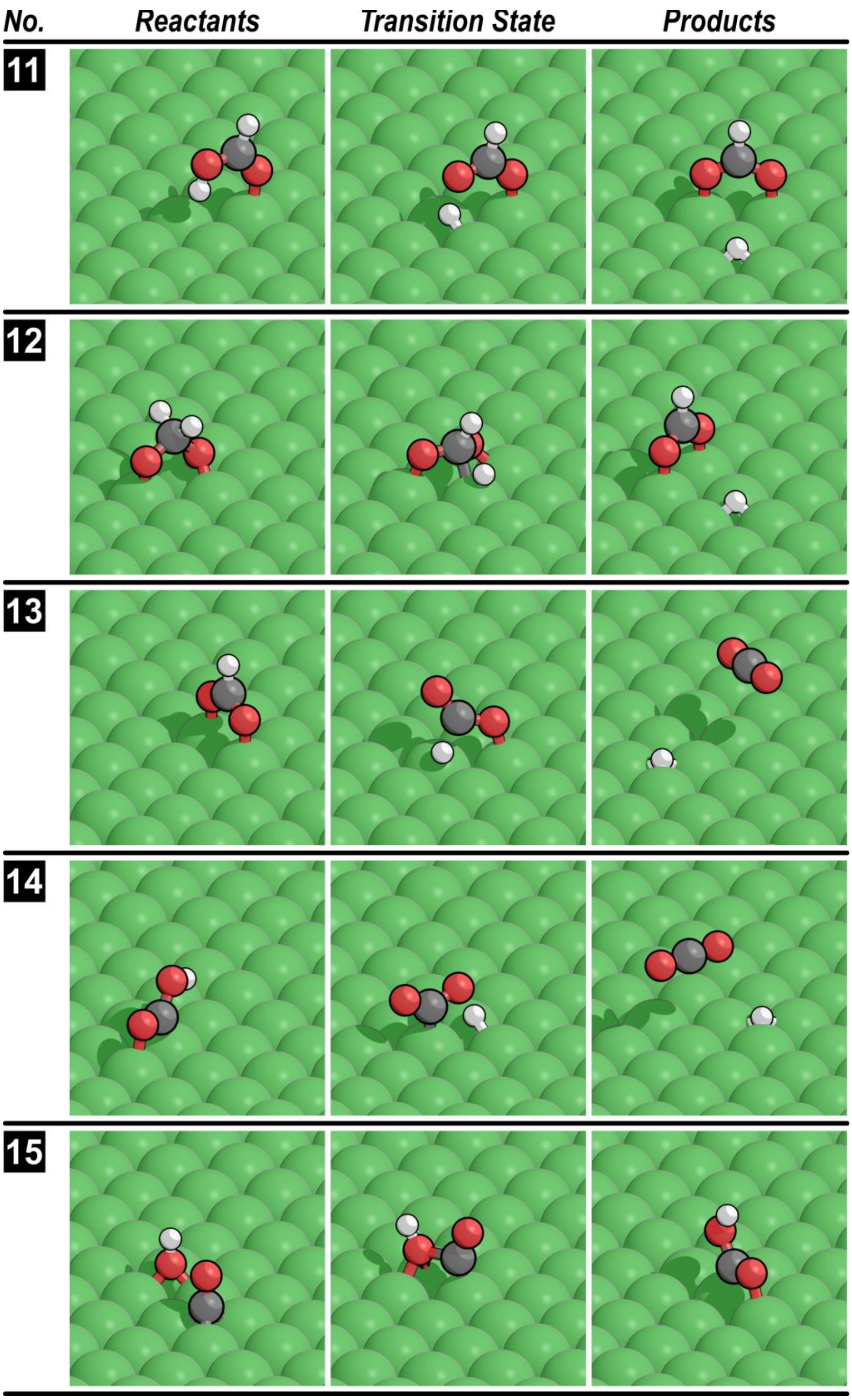

Figure S3 Cont. Reactants, transition state, and products structures for all reactions listed in Table 2 over Ni(111) surface.

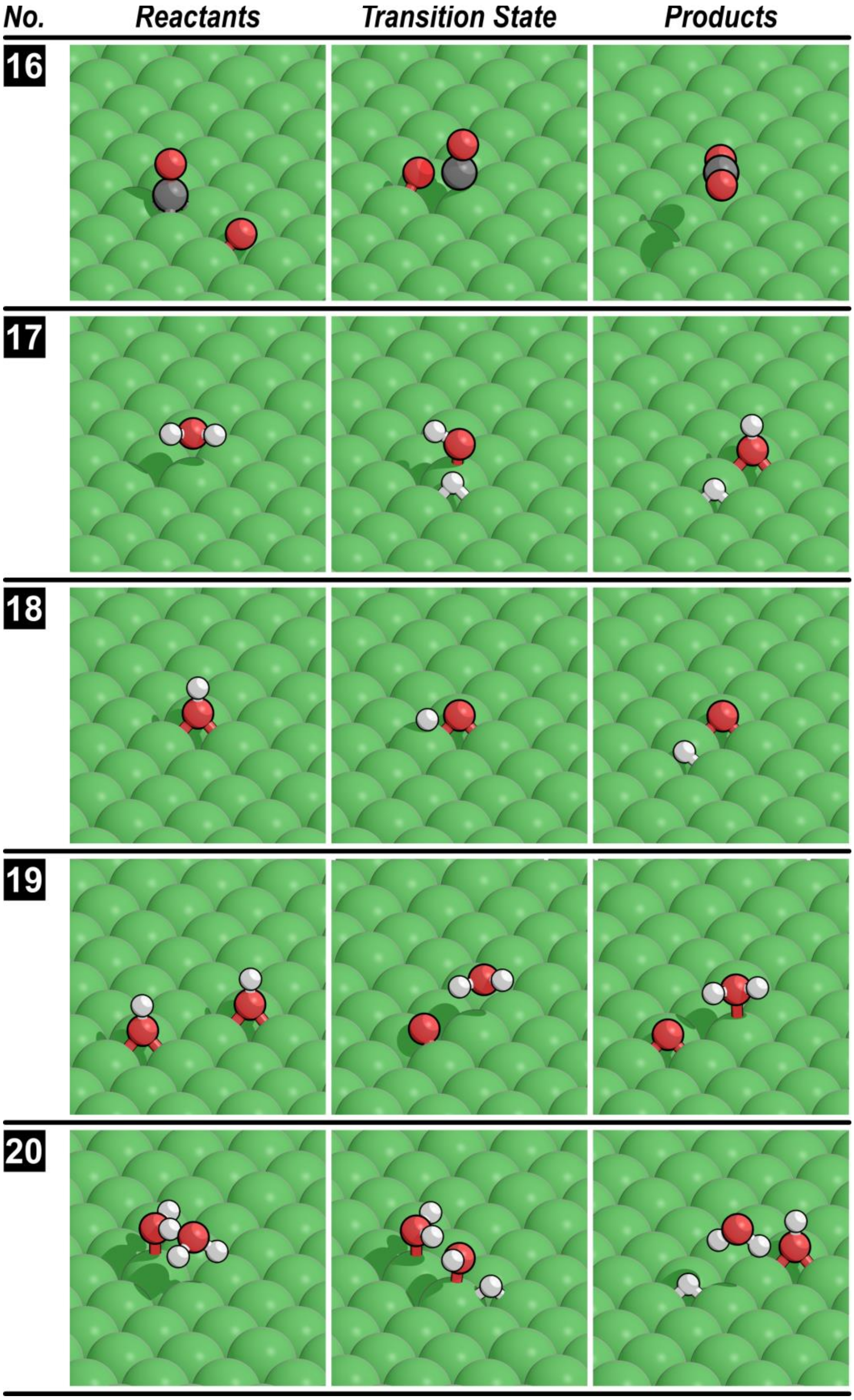

Figure S3 Cont. Reactants, transition state, and products structures for all reactions listed in Table 2 over Ni(111) surface.

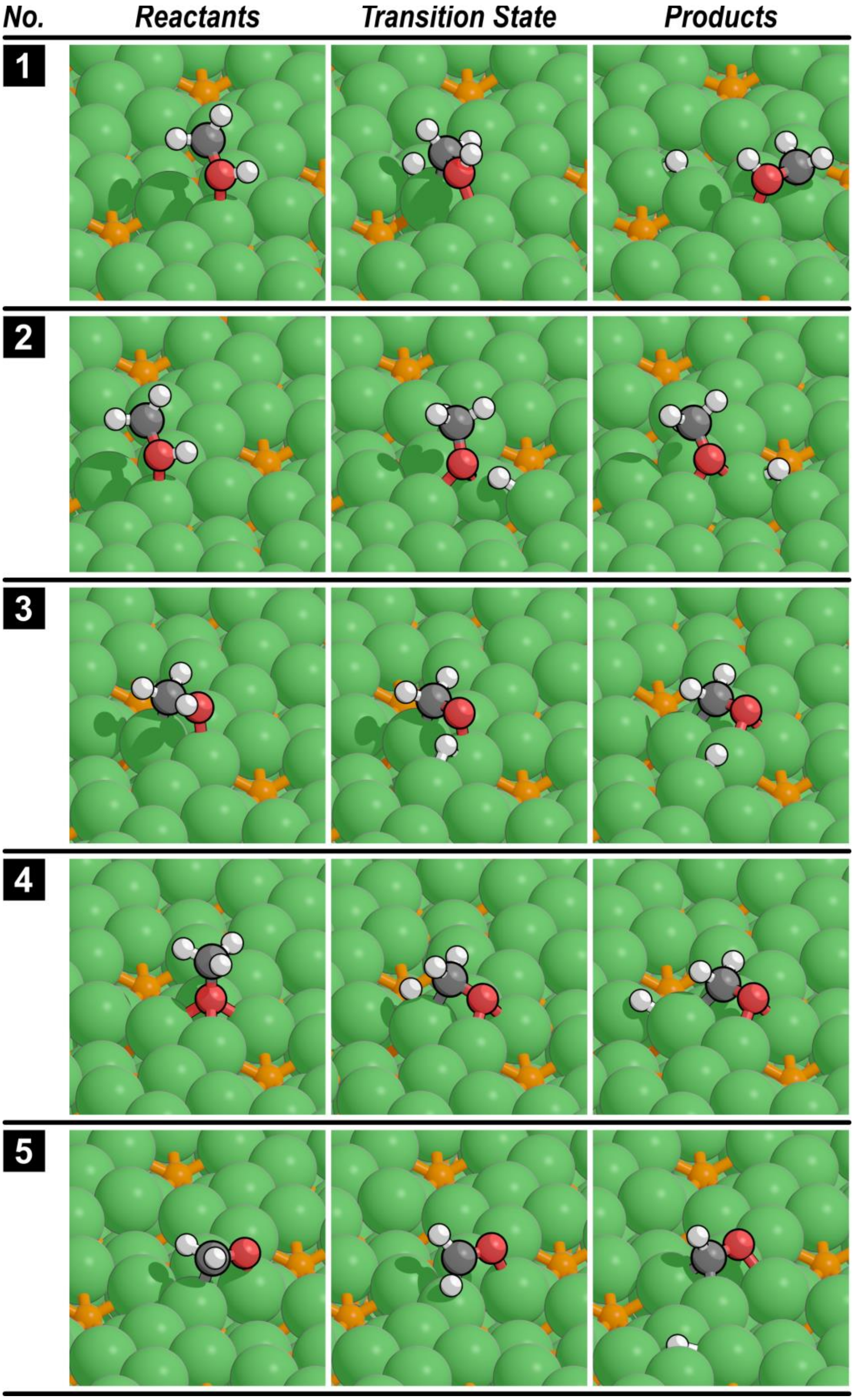

Figure S4. Reactants, transition state, and products structures for all reactions listed in Table 2 over $Ni_{12}P_5(001)$ surface.

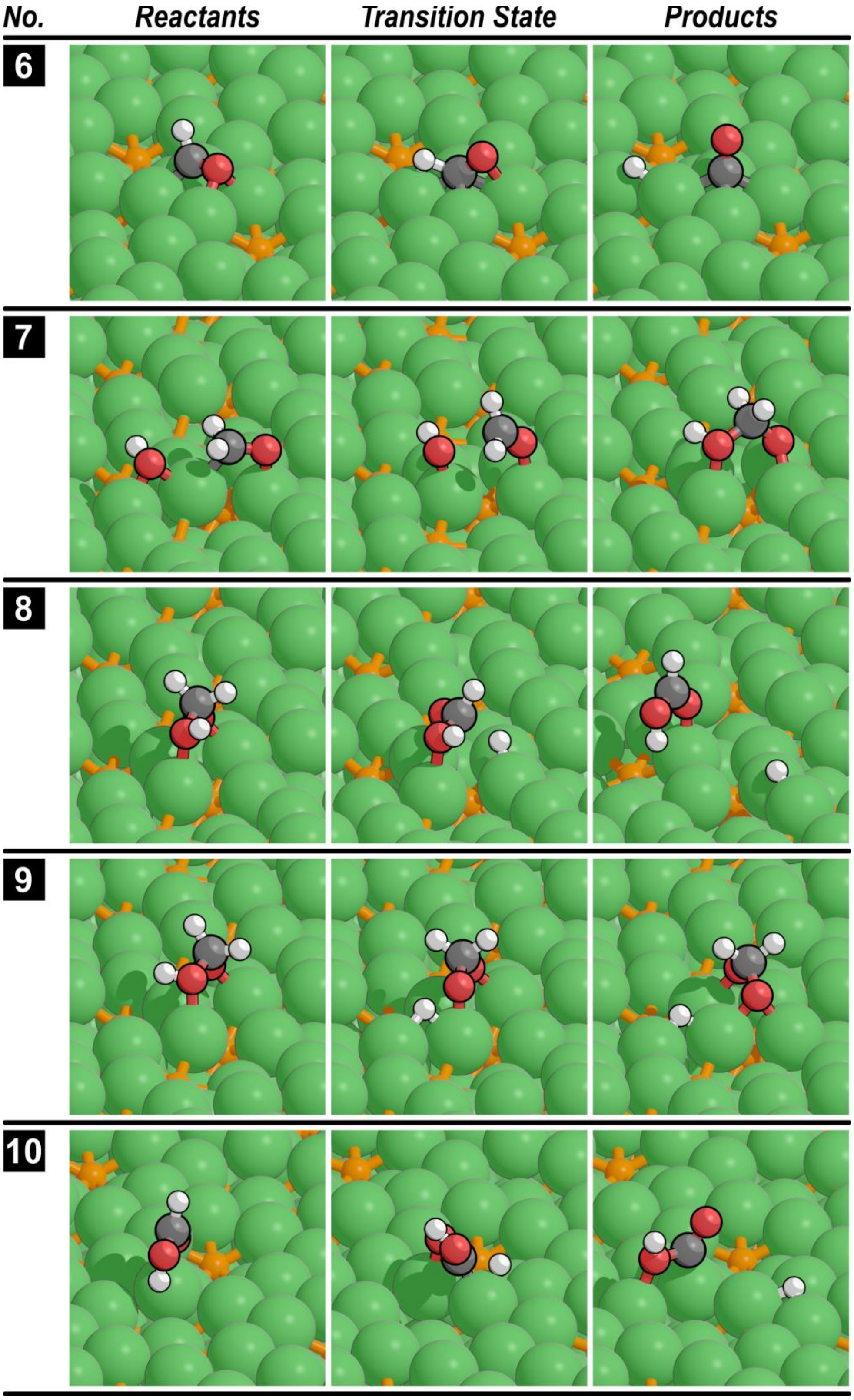

Figure S4 Cont. Reactants, transition state, and products structures for all reactions listed in Table 2 over $Ni_{12}P_5(001)$ surface.

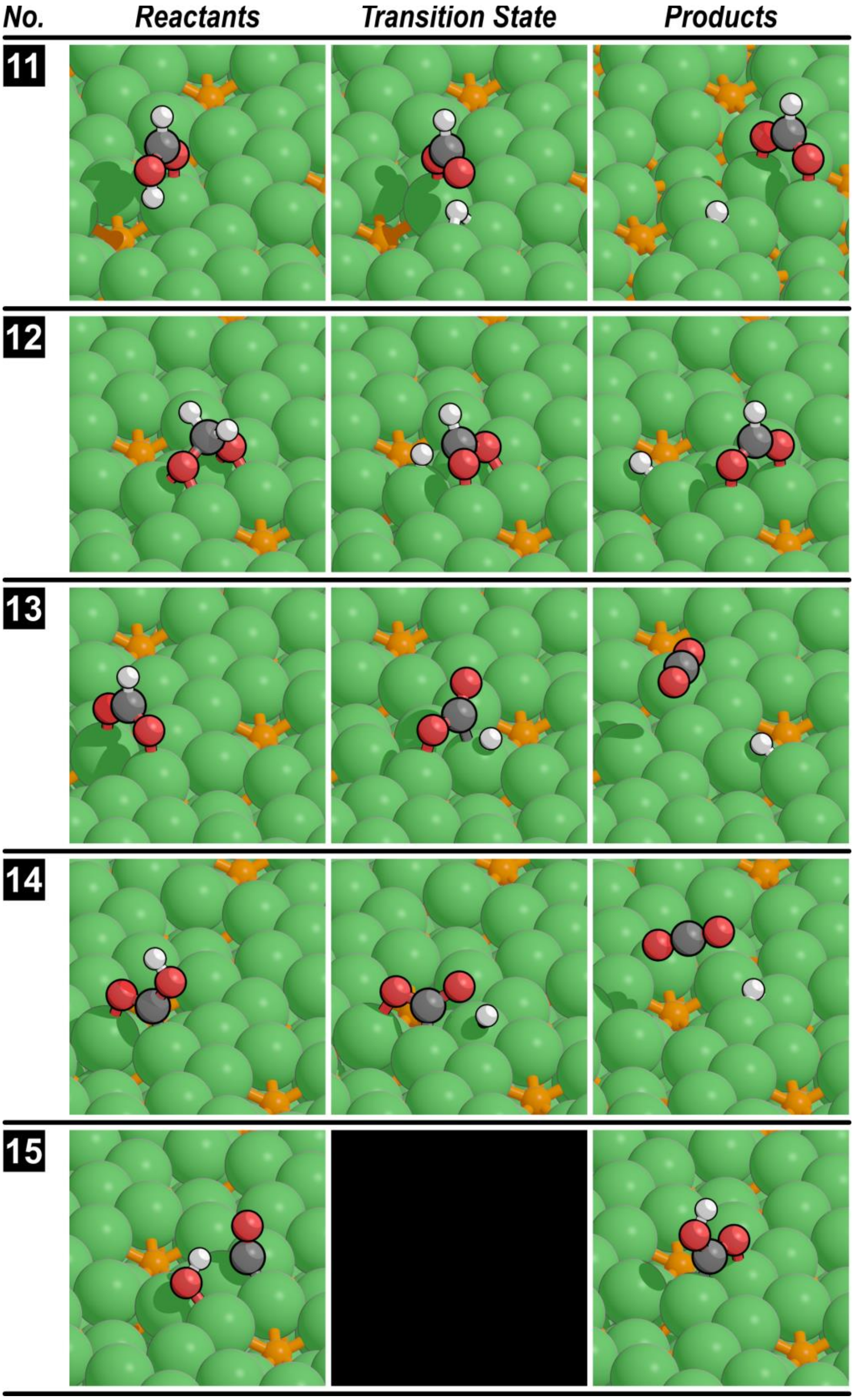

Figure S4 Cont. Reactants, transition state, and products structures for all reactions listed in Table 2 over $Ni_{12}P_5(001)$ surface. No stable transition state was identified for reaction 15.

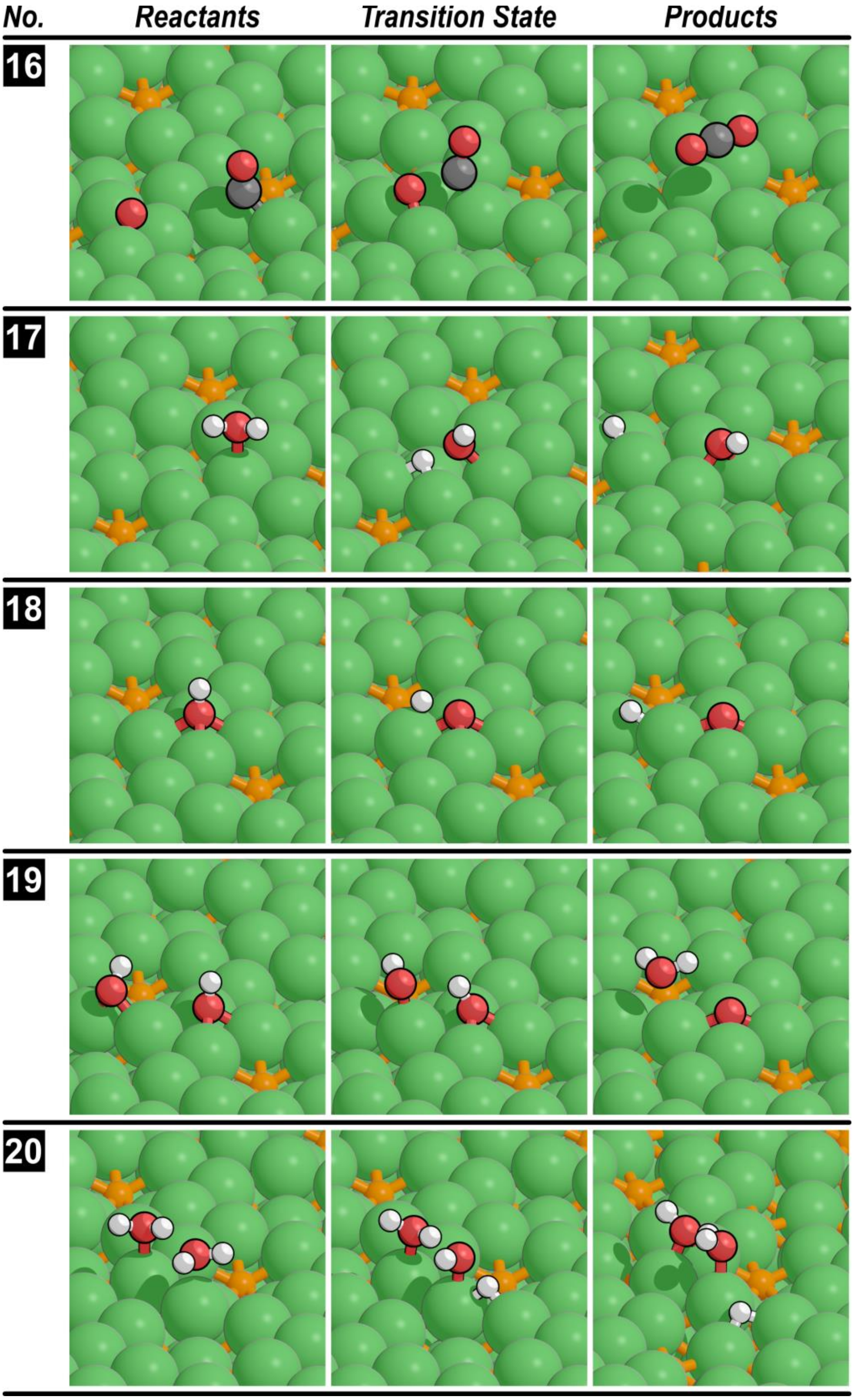

Figure S4 Cont. Reactants, transition state, and products structures for all reactions listed in Table 2 over $Ni_{12}P_5(001)$ surface.

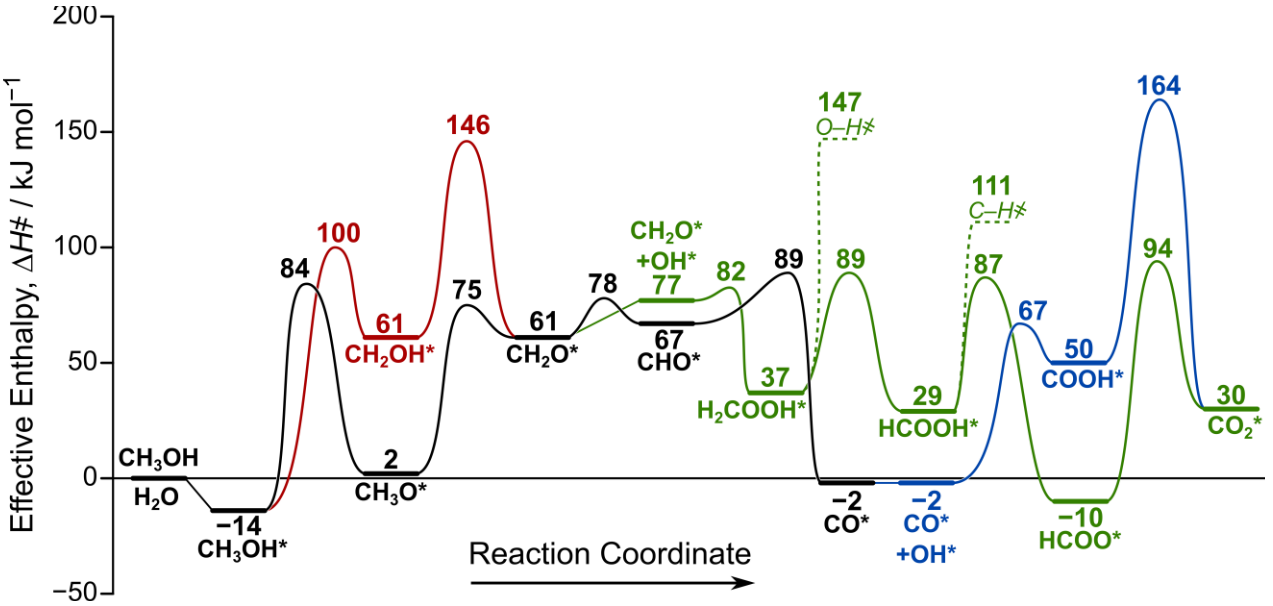

Figure S5. Effective enthalpy diagram for methanol decomposition (black and red), methanol steam reforming (green), and the water–gas shift reaction (blue) pathways on $Ni_2P(001)$ at 573 K. Dehydrogenated H atoms are desorbed from the surface as $H_2(g)$. Dashed lines show barriers for unfavorable routes.
